# Cellular and Molecular Mechanisms of SARS-CoV-2 Spike Protein-Induced Endothelial Dysfunction

**DOI:** 10.3390/cells15030234

**Published:** 2026-01-26

**Authors:** Kelsey C. Muir, Dwight D. Harris, Meghamsh Kanuparthy, Jiayu Hu, Ju-Woo Nho, Christopher Stone, Debolina Banerjee, Frank W. Sellke, Jun Feng

**Affiliations:** 1 Department of Surgery, Division of Cardiothoracic Surgery, Warren Alpert Medical School, Brown University, Providence, RI 02912, USAfrank_sellke@brown.edu (F.W.S.); 2 Cardiovascular Research Center, Division of Cardiothoracic Surgery, Rhode Island Hospital, Providence, RI 02903, USA; 3 Department of Surgery, Division of Cardiothoracic Surgery, University of South Florida, Tampa, FL 33620, USA

**Keywords:** spike protein, SARS-CoV-2, endothelial dysfunction, COVID-19, endothelial permeability

## Abstract

Severe acute respiratory syndrome coronavirus 2 (SARS-CoV-2) infection is initiated by the viral spike proteins, which are key structural components that mediate host cell binding and entry and alter downstream signaling through multiple interactions with endothelial surface receptors. Endothelial dysfunction is a central consequence of COVID-19, contributing to vascular inflammation, barrier disruption, thrombosis, and multi-organ injury affecting the pulmonary, cardiovascular, cerebral, and renal systems. Emerging evidence demonstrates that spike protein-mediated effects, independent of productive viral infection, disrupt endothelial homeostasis through angiotensin-converting enzyme 2 (ACE2) dysregulation, integrin engagement, altered calcium signaling, junctional protein remodeling, oxidative stress, and pro-inflammatory and pro-apoptotic pathways. This review is intentionally focused on spike (S) protein-driven mechanisms of endothelial dysfunction; pathogenic vascular effects attributed to other SARS-CoV-2 structural proteins, including the nucleocapsid (N) protein, are beyond the scope of this discussion. In this review, we synthesize current experimental and translational data detailing the molecular mechanisms by which the SARS-CoV-2 spike protein drives endothelial dysfunction across multiple organ systems and discuss potential therapeutic strategies aimed at preserving endothelial integrity in acute COVID-19 and its long-term vascular sequela.

## 1. Introduction

COVID-19, caused by SARS-CoV-2, was initially characterized as a primary respiratory illness; however, it is now widely recognized as a systemic vascular disease with profound endothelial involvement. Endothelial cells within the microvasculature play a central role in maintaining vascular tone, barrier integrity, hemostasis, and immune regulation across organ systems [1]. Early clinicopathologic studies, including seminal work by Margo and colleagues, revealed widespread complement activation, endothelial injury, and microvascular thrombosis in patients with severe COVID-19, establishing endothelial dysfunction as a possible key driver of disease pathology [2]. Subsequent clinical and experimental evidence has persistently demonstrated that endothelial dysfunction underlies many of the hallmark complications of COVID-19, including acute lung and kidney injury, myocardial injury, cerebrovascular events, and widespread microvascular thrombosis [3,4,5]. These vascular manifestations occur not only during the acute infection but also persist in chronic, long-term sequela of SARS-CoV-2 infections, underscoring the importance of defining the molecular mechanisms driving the endothelial damage [6]. More recent evidence indicates that the severity of acute SARS-CoV-2 infection correlates with the degree of endothelial dysfunction and further suggests that greater endothelial impairment is associated with patients experiencing post-acute sequelae [7,8]. While multiple SARS-CoV-2 structural proteins have been implicated in vascular injury, including evidence supporting the pathogenic role of the nucleocapsid (N) protein, the spike (S) protein has emerged as a critical mediator of endothelial dysfunction, capable of inducing vascular injury independent of productive viral replication.

The spike protein exerts its pathologic effects through multiple converging mechanisms involving angiotensin-converting enzyme 2 (ACE2) dysregulation, integrin engagement, calcium signaling abnormalities, junctional protein disruption, oxidative stress, and activation of pro-inflammatory and pro-apoptotic pathways. Binding of the spike protein to ACE2 not only facilitates viral entry but also disrupts the renin–angiotensin system by promoting ACE2 degradation and shedding and amplifying angiotensin II-driven vascular inflammation [9]. In parallel, spike protein interactions with integrins, mechanosensitive ion channels, and endothelial junctional complexes contribute to barrier disruption, altered intracellular calcium flux, thrombogenic signaling, and endothelial cell death [10,11]. In this review, we comprehensively examine spike protein-mediated endothelial dysfunction across multiple organ systems, integrate emerging evidence from in vitro, in vivo, and translational studies, and highlight shared and organ-specific mechanisms that inform therapeutic strategies aimed at preserving vascular integrity in COVID-19 and its long-term sequelae. Although the present review centers on S protein-driven mechanisms of endothelial dysfunction, accumulating in vitro evidence demonstrates that the N protein has comparable proinflammatory and endothelial-injurious effects [12,13]. In parallel, clinical studies have shown that circulating N antigen levels correlate with COVID-19 disease severity, further implicating N protein as a contributor to vascular pathology beyond spike-mediated signaling, which warrants further review [14].

## 2. Spike Protein-ACE2 Signaling as a Driver of Endothelial Dysfunction in COVID-19

The interaction between the spike protein of SARS-CoV-2 and angiotensin-converting enzyme 2 (ACE2) has profound implications for endothelial dysfunction, a critical aspect of COVID-19 pathology. Endothelial cells express ACE2, which serves as a receptor for the spike protein, facilitating viral entry and subsequent cellular responses that can lead to vascular complications. The binding of the spike protein to ACE2 not only initiates viral infection but also disrupts endothelial barrier integrity, contributing to a cascade of inflammatory responses and vascular dysregulation [9]. ACE2 is a membrane-bound enzyme that is responsible for the processing of angiotensin II into angiotensin-(1–7). ACE2 is expressed across human tissues but in the vascular system is well expressed in vascular endothelial cells and smooth muscle cells [15]. Angiotensin II acts in the cardiovascular system through its AT1 receptor to induce vasoconstriction and inflammation [16]. The action of ACE2 drives a compensatory cardioprotective mechanism that induces vasodilation and anti-inflammatory cascades [17]. However, the spike protein (S protein) induces significant changes in the vasoregulatory environment which may drive endothelial dysfunction.

The S-protein ACE2 interaction may drive SARS-CoV-2 related endothelial dysfunction through two main mechanisms:

1. ACE2 acts as a viral entry receptor and facilitates direct viral endothelial damage.
2. Interaction with the S protein induces ACE2 degradation and shedding leading to downstream pro-inflammatory signaling.

The interaction of the spike protein of SARS-CoV-2 and angiotensin-converting enzyme 2 (ACE2) receptor of host cells is a crucial component involved in viral entry [18]. The S1 subdomain of the trimeric protein contains its receptor moieties which instigate cell entry and require protease activation at the cell boundary to fully enter. This interaction is characterized by a unique conformational change in the spike protein, where multiple receptor binding domains can adopt an “up” position to maximize binding to ACE2, thus enhancing the infectivity of the virus [19]. Recent studies have shown that the binding of the spike protein to ACE2 not only initiates viral entry but also influences the enzymatic activity of ACE2 itself. For instance, the binding can enhance ACE2’s carboxypeptidase activity, which is involved in regulating the renin–angiotensin system, potentially leading to altered physiological responses in infected individuals [20,21,22]. In one study, they demonstrated these effects in a purified enzyme system: recombinant trimeric spike enhances ACE2 catalytic activity (tested at ~0.4–70 μg/mL; commonly 14 μg/mL), supporting a plausible binding-dependent mechanism, although these concentrations may not directly model plasma exposure in patients [20].

It is worth noting that ACE2 activity is classically considered vasculoprotective through degradation of angiotensin II and generation of angiotensin-(1–7). However, spike-induced enhancement of ACE2 carboxypeptidase activity represents a dysregulated, non-physiological state rather than coordinated upregulation of endothelial ACE2 function. Studies demonstrate that spike-ACE2 engagement stabilizes ACE2 in a high-activity conformation while simultaneously promoting receptor internalization and shedding. Additionally, spike protein-mediated increases in ACE2 activity were shown to occur independently of ACE2 protein abundance, vary dramatically across organ systems, and enhance the degradation of multiple vasculoprotective peptides, including apelin 1–13, dynorphin A, and neurotensin, thereby potentially offsetting angiotensin II degradation [20,22]. As a result, enzymatic activity may become uncoupled from normal spatial regulation at the endothelial surface, leading to heterogeneous local angiotensin II/angiotensin-(1–7) signaling. Furthermore, enhanced ACE2 activity may increase the degradation of des-Arg^9^-bradykinin and the cleavage of apelin 1–13, thereby counteracting apelin-mediated nitric oxide-dependent arterial dilation while dysregulating bradykinin-regulated inflammation and endothelial permeability [20,22]. In this context, it appears the enhancement of ACE2 activity occurs alongside receptor internalization, shedding, degradation of vasculoprotective peptides, and downstream inflammatory signaling, collectively promoting endothelial barrier dysfunction and vascular dysregulation despite preserved or transiently increased catalytic activity. Lastly, mutations in both the spike protein and ACE2 can significantly affect their interaction dynamics. Variants of the spike protein, such as D614G and N501Y, have been shown to increase binding affinity to ACE2, which correlates with enhanced transmissibility of the virus [23]. Once the SARS-CoV-2 virus has made entry, replication in the vascular endothelial cells and surrounding smooth muscle result in direct vascular damage and dysfunction.

Although ACE2 is expressed across many cell types, endothelial cells are uniquely susceptible to spike-mediated dysregulation due to their anatomical position and receptor/co-factor landscape at viral entry locations. Spike interactions with endothelial cells may be amplified with co-factors and parallel receptor systems, including heparan sulfate proteoglycans (which facilitate spike engagement and conformational accessibility for receptor interactions) [24] and integrins (notably α5β1), which mediate spike-induced NFκB activation, leukocyte programs, and an inflammatory endothelial phenotype [25]. Moreover, endothelial ACE2 signaling is shaped by protease-dependent regulation of surface ACE2, including TMPRSS2-dependent cleavage and ADAM17-mediated ACE2 shedding, processes that can alter localization, availability, and downstream signaling in a manner that is particularly consequential for vascular homeostasis [26,27].

Downregulation of ACE2 by the S protein may also lead to direct endothelial dysfunction. Lei et al. exposed Syrian hamsters to a pseudovirus expressing S protein, demonstrating in vivo that the lung lysate level of ACE2 was decreased in the infected lungs. These changes were associated with an increased levels of phosphorylated ACE2 and endothelial nitric oxide synthase (eNOS) at sites which downregulate their activity. Lei et al. further incubated the S protein with human pulmonary artery endothelial cells modified with one of two forms of ACE2—ACE2-D with increased stability or ACE2-L with decreased stability. They found increased mitochondrial damage in those cells with the less stable ACE2, indicating that this decreased ACE2 expression may be responsible for increased oxidative stress [28]. The manner by which ACE2 degradation occurs may be driven by increased tumor necrosis factor alpha (TNF-⍺) activation by S-protein, increased a Disintegrin and metalloproteinase 17 (ADAM17) metalloproteinase activity, or by increased ubiquitination and degradation [29,30,31]. Han et al. demonstrate that treatment of human pulmonary endothelial cells incubated with the S protein with bortezomib, an inhibitor of proteasomal activity, prevented the degradation of ACE2 and decreased the expression of serine protease inhibitor PAI-1. PAI has previously been demonstrated to inhibit the conversion of plasminogen to plasmin, preventing the degradation of fibrin clots [32]. Further increased PAI activity has been associated with atherosclerosis and other conditions associated with increased endothelial damage [33,34]. ACE2 degradation also disrupts the balance between Angiotensin II and Angiotensin(1–7) which may tilt the balance towards pro-inflammatory signaling. The presence of increased disassociated and soluble ACE2, increased Ang II, and decreased Ang (1–7) has been well documented in human studies which collected plasma from patients infected with SARS-CoV-2. Decreased Ang (1–7) levels were also associated with increased mortality [35]. Prior research has shown that Ang (1–7) binds to the Mas1 receptor and activates an anti-inflammatory and vasodilatory cascade [17]. Ang (1–7) has also been shown to improve nitric oxide (NO) availability in knockout mice with non-functional ACE2 [36]. Translationally, Ahmed and colleagues demonstrated an association between the downregulation of ACE2 and severe COVID-19 infection as well as post-COVID-19 conditions [37]. While S-protein may directly interact with the vasculature through other modalities, its interaction with ACE2 may be the primary driver of SARS-CoV-2-associated endothelial dysfunction.

Circulating SARS-CoV-2 spike-derived antigens, most commonly the S1 subunit, have been detected in human plasma during acute COVID-19 infection and, in a subset of patients, during post-acute sequelae. One study investigated pediatric patients and found a S protein concentration range of 1.65–1071 pg/mL in acute COVID-19 infection that correlated with disease severity [38]. Other reported plasma concentrations have persistently fallen within the picogram to low nanogram per milliliter range, with higher levels observed in patients with severe disease compared with mild illness and with antigen persistence reported in selected post-acute cohorts [39,40]. Many of the in vitro experimentation utilizes supraphysiologic dosing to determine mechanistic-based effects of the spike protein.

## 3. Integrin-Dependent Effects of the SARS-CoV-2 Spike Protein

Integrins are transmembrane proteins crucial for cell–extracellular matrix adhesion and cell–cell interactions in human cells, impacting processes like signal transduction, migration, proliferation, and survival. Comprised of heterodimeric alpha and beta subunits, integrins form specific receptors with unique binding affinities and functions, transmitting biochemical signals bidirectionally between the extracellular matrix and the cell’s interior. They play vital roles in wound healing, immune responses, and tissue maintenance, helping cells sense and respond to their microenvironment. Abnormal integrin function is linked to diseases such as cancer metastasis, inflammatory conditions (including COVID-19), and fibrosis, making them key targets for therapeutic interventions [41,42,43,44].

Integrins and interactions with spike proteins play an important role in the infection and pathogenesis of COVID-19. Theoretical studies using sequence mapping and computational interaction dynamics have predicted that COVID-19 uses spike protein-to-integrin binding interactions to facilitate cellular infection [45,46]. It has also been further theorized that this interaction could be responsible for other COVID-19 complications, such as endothelial dysfunction and increased platelet activation [45,46].

This theory has been validated by several animal and human cell experiments. Several studies using human cells have shown that αv plays an important role in both viral transmission and pathology. The integrins αvβ3 and αvβ1 helps with COVID-19 invasion, and blocking αvβ3 prevents infection in human lung microvascular endothelial cells [47,48]. Studies using human aortic cells have shown that αvβ3 signaling from COVID-19 triggers the redistribution and internalization of major junction protein VE-Cadherin which leads to the barrier disruption [49,50]. Norris et al. report coating/treatment ranges of S1 from 7.8 to 250 nM and S1-RBD ranges of 10–1000 nM [50].

There is also interest in the role of integrin α5β1 in COVID-19 infection. While it has not been shown that α5β1 facilitates viral entry like αvβ3, α5β1 plays an important role in endothelial and platelet disfunction [25]. The α5β1 integrin has been shown to increase inflammatory response in human endothelial cells and increase vascular leakage in a mouse model [25,51]. Importantly, Biering et al. report these findings when using 50 μg S protein intranasally, which represents a more physiologically relevant route and concentration [51].

Finally, it has been shown in human platelets that integrin α2β3 plays a role in both COVID-19 invasion of platelets as well as potential platelet activation. This possibly helps explain how COVID-19 contributes to increased risk of thrombosis. In summary, integrins play an important part in both the viral transmission and pathophysiology of COVID-19 infection, with αv subtype integrins playing an important role in lung microvascular endothelial cell invasion and α5 and αv subtypes producing endothelial dysfunction [31,48,50,52]. Table 1 provides an overview of the spike protein and differing integrin interactions.

## 4. Spike Protein-Induced Calcium Dysregulation and Ion Channel Signaling in Endothelial Dysfunction

**Figure 1:**
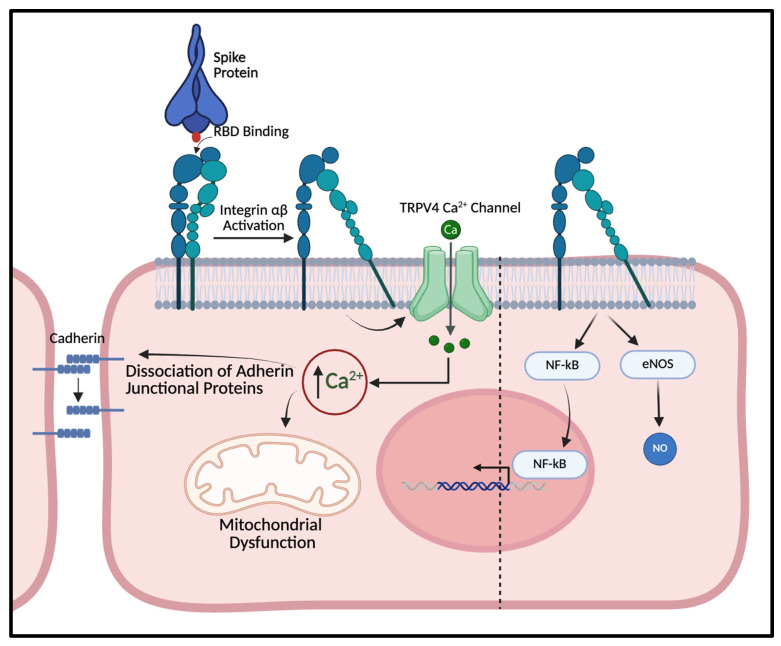
Spike protein–integrin–TRPV4 signaling promotes endothelial dysfunction. Schematic illustrating the proposed mechanisms by which SARS-CoV-2 spike protein disrupts endothelial homeostasis, focusing on integrin and TRPV4 signaling. The S protein binds endothelial integrins via an Arg-Gly-Asp motif called the receptor-binding domain (RBD), triggering integrin activation and downstream opening of the TRPV4 calcium channel. Resultant increases in intracellular Ca^2+^ promote mitochondrial dysfunction, dissociation of adherens junction proteins, activation of pro-inflammatory pathways (particularly nuclear factor kappa B, NFκB), and dysregulation of endothelial nitric oxide synthase (eNOS) and nitric oxide production, culminating in endothelial barrier disruption and endothelial cell death.

Transient Receptor Potential Vanilloid 4 (TRPV4) is a polymodal, nonselective calcium permeable cation channel expressed in vascular endothelium and widely implicated in mechanotransduction, endothelial Ca^2+^ homeostasis, and barrier regulation. Studies consistently demonstrate that the activation of TRPV4 channels affects intracellular calcium levels and endothelial barrier functionality, both in vitro and in vivo [53,54,55,56]. In endothelial cells, TRPV4 can generate localized Ca^2+^ “sparklets” and downstream signaling that couples mechanical or chemical stimuli to changes in vasomotor tone and permeability. Importantly, TRPV4 is functionally linked to integrin-based mechanosensing: mechanical force transmitted through integrins can rapidly activate TRPV4-dependent Ca^2+^ influx, providing a direct pathway by which extracellular cues are converted into intracellular Ca^2+^ signals [57,58]. Depending on vascular bed and signaling context, TRPV4-mediated Ca^2+^ entry can support vasodilation through eNOS and or SK/IK (KCa2.3/KCa3.1) channel activation, while excessive or dysregulated activation promotes cytoskeleton remodeling, junctional destabilization, and increased endothelial permeability [59,60,61]. In the lung, TRPV4 has been repeatedly linked to permeability edema and acute lung injury or ARDS phenotypes, and pharmacologic TRPV4 inhibition (e.g., HC-067047; GSK2193874) improves vascular barrier function and attenuates experimental lung injury in multiple models [62,63,64]. Collectively, these data position TRPV4 as a plausible “common effector” channel through which spike-triggered receptor events can be translated into barrier dysfunction and microvascular injury. Indeed, our preliminary study indicated that spike proteins (S1 and RBD) significantly activated TRPV4 channels, resulting in an increase in Ca^2+^ concentration of human pulmonary endothelial cells subsequently redemonstrated by further studies [65,66] (Figure 1).

Emerging work supports a direct connection between the spike protein receptor binding domain (S-RBD) and endothelial Ca^2+^ dysregulation. In a study, Yang et al. reported that S-RBD induces an acute rise in intracellular free Ca^2+^ concentration ([Ca^2+^]i) consistent with early TRPV4 activation, followed by more sustained Ca^2+^ elevation associated with upregulation of the mechanosensitive Piezo1 and store-operated Ca^2+^ entry (SOCE) component of Orai1 in human pulmonary arterial endothelial cells as well as ACE2 humanized inbred mice [11,66]. Mechanistically, S-RBD-ACE2 engagement promoted formation of channel clusters involving Orai1, Piezo1, and TRPC1, facilitating activation of Piezo1 and SOCE and culminating in endothelial apoptosis; these effects were attenuated by Kobophenol A (blocking S-RBD-ACE2 binding) or intracellular Ca^2+^ chelation (BAPTA-AM) [66]. In vivo, blockade of Piezo1/SOCE signaling with the potent peptide toxin GsMTx4 normalized elevated [Ca^2+^]i and reduced pulmonary microvascular endothelial injury in hACE2 transgenic mice, highlighting Ca^2+^ channel crosstalk as a potentially targetable axis of spike-mediated endothelial damage [11,66]. This important study utilized 4 μg/mL of S-RBD for molecular and functional assessment based on dose-dependent experiments. Together with established integrin-TRPV4 mechanotransduction, these findings raise the possibility that spike-driven receptor engagement converges on TRPV4/Piezo1/SOCE pathways to amplify Ca^2+^ overload, mitochondrial dysfunction, inflammatory signaling, and barrier failure, particularly in pulmonary microvascular endothelium.

Spike protein-induced Ca^2+^ dysregulation may also intersect with other endothelial ion transport pathways relevant to thrombosis and redox stress. Romero et al. demonstrated, using concentrations of RBD from 2 to 5 μg/mL, that SARS-CoV-2 RBD impairs endothelial epithelial sodium channel (ENaC) activity, reduces surface hACE2 expression, and increases reactive oxygen species (ROS) and tissue factor (TF) generation in human lung microvascular endothelial cell monolayers, collectively promoting barrier dysregulation and procoagulant signaling [67]. Notably, the TNF-derived TIP peptide (solnatide/AP301), which directly activates ENaC via the alpha-subunit, mitigated RBD-induced ENaC dysfunction, restored surface hACE2, and reduced ROS/TF generation [67]. These studies broaden the spike-centric view of endothelial injury beyond canonical cytokine signaling and suggest therapeutic stabilization of endothelial ion flux may represent a complementary strategy to preserve endothelial integrity and reduce thrombogenic risk in COVID-19 and related long-term vascular sequelae.

## 5. Spike Protein-Mediated Disruption of Endothelial Junctions and Barrier Function

**Figure 2:**
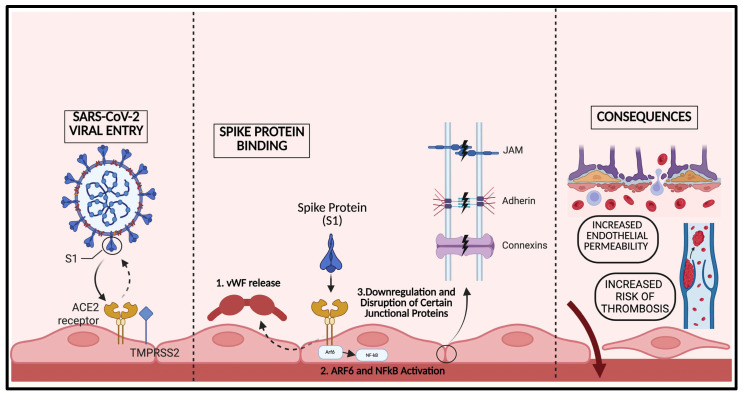
Spike protein-ACE2 disruption of endothelial junctional integrity. Schematic illustrating the spike protein (S1 subunit) binding to the ACE2 receptor on endothelial cells, facilitating viral entry and initiating downstream signaling independent of productive infection. Spike-ACE2 interaction activates ARF6 and NFκB signaling and promotes von Willibrand factor (vWF) release and downregulation and redistribution of key endothelial junctional proteins, including adherens junctions, junctional adhesion molecules (JAMs), and connexins. This disruption results in increased vascular permeability and a heightened risk of thrombosis.

Multiple studies have described SARS-CoV-2 as a “vascular disease” in that it causes a leaky vascular barrier leading to multi-system organ dysfunction [68,69]. Endothelial cells are crucial in maintaining the barrier function between the vasculature and the organ system to which it supplies. There is a complex network of transmembrane “junctional” proteins that maintain the endothelial barrier, including adherens, gap, and tight junctions [10]. Studies have shown the narrow regulation of this protein expression, distribution, and function are key in maintaining the integrity of the vasculature as well as an appropriate immune response after injury [70,71]. Disruption of the expression and location of these proteins can damage the endothelial barrier and have systemic consequences, as seen in the manifestations of SARS-CoV-2 [72,73,74,75,76] (Figure 2).

The main cellular target of SARS-CoV-2 is ACE2, which is ubiquitously expressed on human endothelial cells, by way of its S1 spike protein subunit resulting in viral binding and entry into the cell [77]. Spike protein binding of the ACE2 receptor not only leads to the internalization of SARS-CoV-2, but it also has been shown to trigger multiple downstream molecular consequences manifesting in endothelial dysfunction. Raghavan et al. demonstrated that incubation with solely the spike protein triggered significant downregulation of several crucial junctional proteins, including VE-Cadherin, PECAM-1, JAM-A, and Connexin-43 in healthy and diabetic mouse cerebral arteries [10]. Likely due to a shift in the equilibrium of these junctional proteins, healthy-derived and diabetic-derived microvascular endothelial cells treated with the spike protein had significantly increased endothelial permeability. These effects were determined by co-immunoprecipitation to be in part mediated by increased association of junctional proteins with Rab5a, an endocytic trafficking protein. Interestingly, Agostinis et al. studied viral transfection in pregnant patients and its subsequent effects on endothelial function and permeability. They found stimulation of human umbilical vein endothelial cells (HUVEC’s) with ~10 μg/mL of the S1 subunit had no effect on protein expression of vascular cell adhesion molecule 1 (VCAM-1) and E-selectin; however, it did significantly increase the permeability of endothelial cells within just 15 min [76].

Guo et al. further characterized the mechanisms by which spike proteins confer endothelial barrier breakdown. They concordantly found a time- and dose-dependent increase in endothelial permeability with spike S1 receptor-binding domain (RBD) [68]. Additionally, they determined an increase in endothelial permeability occurred concurrently with increased secretion of von Willibrand Factor, in which both were mediated via binding of ACE2 receptor and subsequent ADP-ribosylation factor (ARF)6 activation. ARF6 is a Ras-related small GTPase that has previously been shown to induce leaky vasculature in LPS-induced sepsis, which in a similar manner confers spike protein endothelial barrier dysfunction. Finally, they showed selective inhibition of ARF6 using chlortetracycline significantly inhibited endothelial permeability and nuclear factor kappa B (NF-kB) signaling, but did not inhibit the mammalian target of rapamycin (mTOR) or dynamin pathways. Further elucidation of the downstream effects of spike proteins was determined by Biancatelli et al. utilizing a unique model of K18-hACE2 transgenic mice that overexpress human ACE2 receptors. This study found that intratracheal instillation of the S1 spike protein, and not intact spike protein, induced severe acute lung injury due to activation of NF-kB and signal transducer and activator of transcription 3 (STAT3) pathways. To determine the cause of alveolar inflammation and edema, the study demonstrated that the S1 subunit of the spike protein, and again not the intact spike protein, produced a significant decrease in transendothelial resistance (TER) in a dose-dependent manner from 1 to 10 μg/mL, which indicated an increase in permeability and dysfunction of the endothelial barrier [78]. Multiple studies have confirmed similar pathways by which the spike protein and its RBD result in endothelial barrier dysfunction via integrin, transforming growth factor beta (TGFβ) and NFκB signaling changes [49,79]. Endothelial barrier disruption is convincingly a result of the spike protein S1 subunit binding to the ACE2 receptor, leading to a multitude of downstream effects on cell trafficking, structural and junctional protein expression, and inflammation. However, further work by Shahoha et al. demonstrated multiple other viral antigens that may confer or work in synergy with spike proteins to disrupt the endothelial cell barrier. Their group cloned and expressed 26 of the individual proteins of SARS-CoV-2 to characterize their effect on endothelial barrier integrity of HUVEC’s [80]. Specifically, nonstructural protein (nsp)2, nsp5_c145a, and nsp7 induced significant changes in endothelial permeability by affecting the expression and function of tight junction proteins cadherin-5, ZO-1, and β-catenin.

Meanwhile the S1 subunit of the spike protein appears to have the most detrimental effects on the endothelial barrier, primarily via ACE2 binding; there are likely multiple complex pathways in which it induces endothelial permeability and other viral antigens that further heighten this effect. Additional studies have modeled the human blood–brain barrier (BBB) as one of the most important human vascular barriers to elucidate the likely similar effects of spike proteins and understand the mechanistic determinants resulting in the neurological effects of SARS-CoV-2. T.P. Buzhdygan et al. utilized an innovative 3D BBB model from polymerizing hydrogels and demonstrated that various spike proteins, including the subunit S1, subunit 2, and the RBD, all significantly reduced the electrical resistance (an analytical means to examine barrier “tightness”) and increased paracellular permeability (barrier “leakiness”) in a dose-dependent manner [81]. This was further corroborated by the work of DeOre, using a similar 3D BBB microfluidic model to show disruption of the endothelial barrier by spike protein binding via the upregulation of ACE2 receptor and activation of RhoA, which is a key regulator of endothelial cytoskeleton and tight junction complexes [82]. However, the former study found two additional mechanistic determinants by which spike protein interaction with cerebral endothelial cells result in barrier disruption: an ACE2 independent point of contact and the upregulation of cell adhesion molecules (CAMs), matrix metalloproteinase (MMPs), and pro-inflammatory cytokines.

## 6. Spike Protein-Driven Inflammatory and Apoptotic Signaling in Endothelial Dysfunction

Infection with SARS-CoV-2 induces a hyperactive immune response, endothelial injury, and hypercoagulable state. The SARS-CoV-2 S protein specifically has been detected as an isolated viral element in human tissue reservoirs and serves as a target for mRNA COVID-19 vaccines. A recent study showed that 1–5 μg/mL of S protein serves as both a pro-inflammatory and pro-thrombotic signal, stimulating human endothelial cells and monocytes through activation of NFκB and subsequent cytokine release, resulting in priming and activation of NLRP3 inflammasome and production of mature pro-inflammatory IL-1β in both cell types [83]. In parallel, IL-1β stimulates prothrombotic cascades via production of vWF and factor VIII/tissue factor without an attendant increase in antithrombotic pathways mediated by ADAMTS-13, which most potently counteracts procoagulant activity of vWF multimers. TLR4 receptors mediate these effects in monocytes, but not in endothelial cells. Several studies have investigated pathway activation that underlies changes in endothelial cell function following SARS-CoV-2 infection. Subunit 1 of the S protein (S1), which contains the receptor binding domain of ACE2, may mediate pulmonary endothelial dysfunction through mechanisms independent of ACE2 enzymatic activity and viral replication [9].

One mechanism may occur via amplification of anaphylatoxin C3 and C3a receptor (C3aR) expression, leading to lung vascular fibrinogen/platelet aggregation and diffuse alveolar damage [84]. C3aR antagonism may be a potential therapeutic strategy for limiting S1-dependent lung pathology. Early loss of pulmonary endothelial thromboresistance through decreased thrombomodulin and increased vWF has also been observed. S1 has also been associated with increased production of IL-6, MCP-1, ICAM-1, and PAI-1 and upregulation of NFκB via ACE2 to induce endotheliitis [9]. Lectin pathway recognition molecules of the complement system, including MBL, FCN-2, and CL-11, bind the S protein and lead to subsequent C3b and C4b deposition via action of effector enzyme MASP-2 [2,12]. This was additionally observed with the N protein. Mannan-binding lectin-associated serine protease 2 (MASP-2) inhibitor Narsoplimab has shown promise in recent clinical trials studying the treatment of severe COVID-19 infection [85]. Alternative mechanisms inducing similar endothelial dysfunction also involve the N protein and viroporin. Generalized endotheliitis may in turn drive intense platelet activation through release of the platelet-derived calcium-binding proteins SA100A8 and SA100A9 and capitulate cardiopulmonary complications [86]. Host cell proteases, TMPRSS2 and histone acetyltransferases that are recruited by SARS-CoV-2 to cleave ACE2 S protein and facilitate virus entry, also represent viable drug targets for limiting pro-inflammatory and pro-thrombotic effects [87].

The S protein, responsible for allowing SARS-CoV-2 virus to enter the cell, has two subunits: S1, which binds to ACE2 located on the cell’s surface, and S2, which is digested by transmembrane serine protease 2 in order for the virus to fuse into the membrane. Subsequently, rapid replication by the virus triggers an excessive inflammatory response, hypercytokinemia, complement activation, and macrophage activation, which in turn leads to endothelial dysfunction [88]. Localization of the virus to the mitochondria impedes its function and generates mitochondrial reactive oxygen species, causing oxidative stress and cellular damage in turn [89]. Interestingly, numerous studies have revealed that the structural proteins of SARS-CoV-2 virus can independently damage the lung, even without infection by the virus itself.

Notably, the S protein has been shown to elevate proinflammatory and proapoptotic signaling in vitro through multiple pathways. The S protein activates the NOD-like receptor family pyrin domain containing 3 (NLRP3) inflammasome of the NLR family pyrin domain, leading to higher levels of IL-1β and IL-18 and indication of inflammation and apoptosis [88]. Additionally, the S protein facilitates polarization of M1 macrophages, thus triggering apoptosis and generating reactive oxygen species. A recent study delved deeper into the mechanism behind SARS-CoV-2 spike promotion of apoptosis. Autophagy, in which lysosomes degrade cellular materials due to ER stress and subsequent protein unfolding, is a cellular defense mechanism that can cause apoptosis and in more extreme cases, death of the cell. The spike protein increases autophagy in cells with ACE2 through the PAM (PI3K/AKT/mTOR) signaling pathway, subsequently triggering tightly interlinked inflammatory and apoptotic responses [90]. Pro-inflammatory cytokines such as TNF-α, IL-6, IL-8, and IL-1β were reported, and apoptosis was quantified through Western blotting of a decrease in Bcl-2 and increase in Bax [90]. Additionally, the spike protein lowers levels of ACE2 and TMPRSS2 while increasing NOX2 and NOX4 levels, and the tight junction proteins and reduced nitric oxide causes cellular dysfunction following oxidative stress and apoptotic responses [91]. These pathological responses of inflammation and vascular dysregulation are closely related to thrombosis, with the tissue factor being shown to be prothrombotic [92].

Diabetes increases susceptibility to SARS-CoV-2 complications and adds nuance to complex causes of comorbidity in patients. Specifically, high glucose levels similarly reduce ACE2 and activate NOX2 and NOX4 but do not impact TMPRSS2 [91]. Thus, endothelial cell injury in hyperglycemic SARS-CoV-2 patients seem to utilize the ACE2-NOX axis of the S protein. The spike protein further worsens inflammation and endothelial cell death in diabetic patients, doing so through the Toll-like receptor (TLR) signaling pathway and the renin–angiotensin–aldosterone system [93,94]. Qian et al. supports that the mitogen-activated protein kinase mechanism, on top of the TLR2/NF-κB pathway, is responsible for the nucleocapsid protein activation of human endothelial cells [93]. Simvastatin was found to significantly inhibit the nucleocapsid protein activation, showcasing its potential to mitigate the effect of SARS-CoV-2 in diabetic patients [93].

In vivo, Lei et al. demonstrate the cardioprotective role of ACE2 and that the S protein by itself can downregulate ACE2, leading to vascular endothelial cell damage. During the entry of SARS-CoV-2, S1 competes with Angiotensin II to bind with ACE2; S1-ACE2 binding prevents further ACE2 activity and causes downregulation of ACE2 [28]. ACE2 expression is also controlled by AMP-activated protein kinase (AMPK) and murine double minute 2 (MDM2) interactions, which phosphorylate and ubiquitinate the receptor, respectively. Syrian hamsters exposed to a S protein-containing pseudovirus saw significant lung damage; lower levels of phospho-AMPK, phospho-ACE2, and ACE2; higher levels of MDM2; and impaired acetylcholine-induced, endothelium-dependent vasodilation of pulmonary arteries [28]. These results, despite being limited by the utilization of a pseudovirus lacking infectious capability, provide support for the idea of S protein-related redox stress decreasing AMPK, increasing MDM2, and destabilizing ACE2 to impair the renin–angiotensin system and worsen endothelial dysfunction [28]. Moreover, they are significant in demonstrating endothelial injury caused by the S protein. The S1 subunit demonstrates localization to the microvessel endothelium of mice brains, along with spatial overlap with caspase-3, ACE2, IL6, TNFα, and C5b-9. Even by itself, the S1 subunit of the spike protein activates MEK/ERK, which has been established as a pathway for cell growth and supports replication of viruses [95,96]. SARS-CoV-2 patients have thickened pulmonary vascular walls, a detrimental effect that may be caused by S1-induced cellular growth [95]. Therefore, the SARS-CoV-2 spike protein alone, without the other structural, accessory, and non-structural viral components, is able to induce cell signaling in vascular endothelial cells both in vivo and in vitro [84,95,96]. Vaccinations utilizing an antibody to combat S protein activity are highlighted as a potential method to protect against SARS-CoV-2-related endothelial dysfunction.

## 7. Conclusions and Future Directions

Collectively, the evidence reviewed herein establishes endothelial dysfunction as a unifying and central feature of COVID-19 pathophysiology, with the S protein serving as one of the important molecular drivers of microvascular injury across organ systems. Beyond its canonical role in viral entry, the spike protein exerts pleiotropic effects on endothelial cells through ACE2 dysregulation, integrin interaction, aberrant calcium signaling, junctional protein dysregulation, oxidative stress, and activation of pro-inflammatory, pro-thrombotic, and pro-apoptotic pathways. Importantly, many of these effects occur independently of active viral replication and infection, supporting the concept that COVID-19 represents, at least in part, a spike protein-mediated vascular disease. These mechanisms converge to impair endothelial barrier integrity, alter vasoregulatory signaling, and promote thrombosis, thereby contributing to pulmonary, cardiac, cerebral and renal dysfunction observed in both acute disease and chronic sequela after infection.

Of note, although several COVID-19 vaccines encode or deliver the SARS-CoV-2 spike protein, spike biology in vaccination differs fundamentally from that during infection. Vaccine-derived spike expression is non-replicative and prefusion-stabilized [97]. Large clinical studies have not shown a link between vaccination and generalized endothelial dysfunction, and rare vascular adverse events appear immune-mediated and far less frequent than vascular complications seen with SARS-CoV-2 infection [98,99]. However, there is emerging evidence that demonstrates the SARS-CoV-2 mRNA vaccine-derived spike protein can persist for weeks to months after vaccination, be detected in multiple organ systems including the heart and cerebral arteries, and engage overlapping biochemical pathways with those implicated in the pathogenic effects of virus-derived spike protein discussed throughout this review [100,101,102,103]. Accordingly, further therapeutic investigation of these pathways and persistence of the vaccine spike protein may help inform approaches to mitigate post-acute sequelae of SARS-CoV-2 infection and vaccine-associated adverse effects.

Despite substantial progress, several critical knowledge gaps remain. Future studies should focus on defining the relative contributions of ACE2-dependent versus ACE2-independent S protein signaling across distinct vascular beds, elucidating how spike–integrin and mechanosensitive ion channel interactions integrate with endothelial calcium dynamics, and determine how host factors such as metabolic syndrome, aging, and sex influence endothelial vulnerability. Greater emphasis on human-relevant models, such as organ-on-chip systems and vascularized organoids, will be essential to translate mechanistic insights into clinical relevance. Therapeutically, targeting spike protein-mediated endothelial dysfunction represents a promising avenue to mitigate both acute COVID-19 infection and ideally long-term vascular sequela. For example, soluble recombinant ACE2 can neutralize spike and has been shown to reduce SARS-CoV-2 infection in engineered human blood vessel organoids, providing a mechanistic rationale for attenuating both viral burden and vascular injury [104]. This was also demonstrated using the commonly available drug acetylsalicylic acid (ASA), which impaired spike binding with ACE2 and markedly reduced lung injury and inflammation in a small animal model [105]. Continued investigation into spike protein-specific interventions as well as the comparison between the S protein and N protein mechanisms and interventions may ultimately refine strategies to preserve endothelial health in current and future coronavirus-related diseases.

## Figures and Tables

**Table 1:** Experimental evidence of spike protein and integrin interactions.

Author	Integrin	Model	Finding
Bugatti et al., 2022 [47]	αvβ3	Human lung microvascular endothelial cells	The authors found that pretreatment of virus with αvβ3 integrin or pretreatment of cells with a monoclonal antibody against αvβ3 integrin inhibited SARSCoV-2 entry into human lung microvascular endothelial cells.
Bugatti et al., 2023 [44]	αvβ3	Human lung microvascular endothelial cells	The authors found that the D405N mutation inhibits Omicron BA.5 infection of HL-mECs and their dysfunction because of the lack of spike/integrins interaction.
Nader et al., 2022 [49]	αvβ3	Human Aortic Endothelial Cells	The authors found that COVID-19 interaction with integrin αvβ3 expressed on human endothelial cells triggers the redistribution and internalization of major junction protein VE-Cadherin which leads to the barrier disruption.
Cai et al., 2022 [48]	αvβ1	Human embryonic kidney, human colon cancer, human ovarian cancer, human breast cancer, human liver cancer, human pancreatic cancer, human gastric cancer, human normal lung epithelial cells, human lung adenocarcinoma cells, and mouse embryonic fibroblast cells	The authors found that αvβ1 was highly enriched in COVID-19. Further studies using mouse cells expressing human suggest that integrin αvβ1 was unable to function as an independent receptor but could significantly facilitate the cellular entry of COVID-19.
Zhang et al., 2023 [25]	α5β1	Human endothelial cells	The authors found that α5β1 interaction increased secretion of proinflammatory cytokines IL-6 and IL-1β.
Biering et al., 2022 [51]	α5β1	Mice	The authors found that glycosaminoglycans, integrins, and the TGF-β signaling are required for S-mediated barrier dysfunction. COVID-19 infection caused leak in vivo, which was reduced by inhibiting integrins.
Norris et al., 2023 [50]	αvβ3, αvβ6, α5β1	Mouse embryos	The authors found direct binding of S1-RBD to recombinant human αvβ3 and αvβ6 integrins, but not α5β1 integrins, using surface plasmon resonance. S1-RBD stimulated tyrosine phosphorylation of the adhesion mediators FAK, Src, and paxillin; triggered Akt activation; and supported cell proliferation.
Ito et al., 2023 [52]	α2β3	Human platelets	The authors found that SARS-CoV-2 entered platelets through association of S-protein with αIIbβ3. COVID-19 may induce phenotypes such as granule secretion and shape change in platelets due to platelet activation.

Summary of several experimental studies examining SARS-CoV-2 spike protein interactions with integrins and their subsequent effects on endothelial and platelet function. The table highlights the integrin subtype implicated in S protein binding, the experimental model utilized, and key findings.

## Data Availability

No new data were created or analyzed in this study. Data sharing is not applicable to this article.

## Abbreviations

The following abbreviations are used in this manuscript:

ACE2	Angiotensin-converting enzyme 2
ADAM17	A Disintegrin and Metalloproteinase 17
AMPK	AMP-activated protein kinase
Ang II	Angiotensin II
Ang-(1–7)	Angiotensin-(1–7)
ARDS	Acute respiratory distress syndrome
ARF6	ADP-ribosylation factor 6
AT1	Angiotensin II type 1 receptor
BAPTA-AM	1,2-bis(o-aminophenoxy)ethane-N,N,N′,N′-tetraacetic acid acetoxymethyl ester
BBB	Blood–brain barrier
CAMs	Cell adhesion molecules
COVID-19	Coronavirus disease 2019
eNOS	Endothelial nitric oxide synthase
ENaC	Epithelial sodium channel
ER	Endoplasmic reticulum
FAK	Focal adhesion kinase
GsMTx4	GsMTx4 peptide toxin (mechanosensitive channel inhibitor)
HC-067047	TRPV4 antagonist
HL-mECs	Human lung microvascular endothelial cells
HL-MVEC	Human lung microvascular endothelial cells
HUVECs	Human umbilical vein endothelial cells
IK	Intermediate-conductance Ca^2+^-activated K^+^ channel (KCa3.1)
IL-1β	Interleukin-1 beta
IL-6	Interleukin-6
IL-8	Interleukin-8
IL-18	Interleukin-18
JAM	Junctional adhesion molecule
JAM-A	Junctional adhesion molecule A
K18-hACE2	Keratin 18 promoter–driven human ACE2 transgenic
KCa2.3	Small-conductance Ca^2+^-activated K^+^ channel 3
KCa3.1	Intermediate-conductance Ca^2+^-activated K^+^ channel 1
Mas1	Mas receptor (Mas1 proto-oncogene receptor)
MDM2	Mouse double minute 2 homolog
MEK/ERK	Mitogen-activated protein kinase/Extracellular signal-regulated kinase
MMPs	Matrix metalloproteinases
mTOR	Mechanistic target of rapamycin
NF-κB	Nuclear factor kappa B
NO	Nitric oxide
NOX2	NADPH oxidase 2
NOX4	NADPH oxidase 4
nsp	Nonstructural protein
Orai1	ORAI calcium release-activated calcium modulator 1
PAI-1	Plasminogen activator inhibitor-1
PAM	PI3K/AKT/mTOR pathway
PECAM-1	Platelet endothelial cell adhesion molecule-1
Piezo1	Piezo-type mechanosensitive ion channel component 1
PI3K	Phosphoinositide 3-kinase
RBD	Receptor-binding domain
RGD	Arginine–glycine–aspartic acid
RhoA	Ras homolog family member A
ROS	Reactive oxygen species
S protein	Spike protein
S1	Spike subunit 1
S2	Spike subunit 2
SARS-CoV-2	Severe acute respiratory syndrome coronavirus 2
SK	Small-conductance Ca^2+^-activated K^+^ channel
SOCC	Store-operated calcium channel(s)
SOCE	Store-operated calcium entry
STAT3	Signal transducer and activator of transcription 3
TF	Tissue factor
TGF-β	Transforming growth factor beta
TIP peptide	TNF-derived TIP peptide (Solnatide/AP301)
TLR	Toll-like receptor
TMPRSS2	Transmembrane serine protease 2
TNF-α	Tumor necrosis factor alpha
TRPC1	Transient receptor potential canonical 1
TRPV4	Transient receptor potential vanilloid 4
TER	Transendothelial electrical resistance
vWF	von Willebrand factor
VE-cadherin	Vascular endothelial cadherin
ZO-1	Zonula occludens-1
